# Highly tailorable gellan gum nanoparticles as a platform for the development of T cell activator systems

**DOI:** 10.1186/s40824-022-00297-z

**Published:** 2022-09-30

**Authors:** Daniel B. Rodrigues, Helena R. Moreira, Mariana T. Cerqueira, Alexandra P. Marques, António G. Castro, Rui L. Reis, Rogério P. Pirraco

**Affiliations:** 1 3B’s Research Group, I3Bs – Research Institute on Biomaterials, Biodegradables and Biomimetics, University of Minho, Headquarters of the European Institute of Excellence on Tissue Engineering and Regenerative Medicine, AvePark, Parque de Ciência e Tecnologia, Zona Industrial da Gandra, Barco, 4805-017 Guimarães, Portugal; 2 ICVS/3B’s – PT Government Associate Laboratory, 4805-017 Braga/Guimarães, Portugal; 3 School of Medicine, Life and Health Sciences Research Institute (ICVS), University of Minho, Braga, Portugal

**Keywords:** Nanoparticles, Gellan gum, T cell stimulation, Cytotoxic T cells

## Abstract

**Background:**

T cell priming has been shown to be a powerful immunotherapeutic approach for cancer treatment in terms of efficacy and relatively weak side effects. Systems that optimize the stimulation of T cells to improve therapeutic efficacy are therefore in constant demand. A way to achieve this is through artificial antigen presenting cells that are complexes between vehicles and key molecules that target relevant T cell subpopulations, eliciting antigen-specific T cell priming. In such T cell activator systems, the vehicles chosen to deliver and present the key molecules to the targeted cell populations are of extreme importance. In this work, a new platform for the creation of T cell activator systems based on highly tailorable nanoparticles made from the natural polymer gellan gum (GG) was developed and validated.

**Methods:**

GG nanoparticles were produced by a water in oil emulsion procedure, and characterized by dynamic light scattering, high resolution scanning electronic microscopy and water uptake. Their biocompatibility with cultured cells was assessed by a metabolic activity assay. Surface functionalization was performed with anti-CD3/CD28 antibodies via EDC/NHS or NeutrAvidin/Biotin linkage. Functionalized particles were tested for their capacity to stimulate CD4^+^ T cells and trigger T cell cytotoxic responses.

**Results:**

Nanoparticles were approximately 150 nm in size, with a stable structure and no detectable cytotoxicity. Water uptake originated a weight gain of up to 3200%. The functional antibodies did efficiently bind to the nanoparticles, as confirmed by SDS-PAGE, which then targeted the desired CD4^+^ populations, as confirmed by confocal microscopy. The developed system presented a more sustained T cell activation over time when compared to commercial alternatives. Concurrently, the expression of higher levels of key cytotoxic pathway molecules granzyme B/perforin was induced, suggesting a greater cytotoxic potential for future application in adoptive cancer therapy.

**Conclusions:**

Our results show that GG nanoparticles were successfully used as a highly tailorable T cell activator system platform capable of T cell expansion and re-education.

**Supplementary Information:**

The online version contains supplementary material available at 10.1186/s40824-022-00297-z.

## Introduction

Many advances have been made in the field of immunotherapy with the introduction of adoptive T cell therapy and CAR T cell therapies. These consist in the administration of either autologous T cells primed and expanded *in vitro* or genetically modified T cells that encode for transmembrane chimeric molecules with the capacity to both recognize tumor surface antigens and trigger cytotoxic machinery specifically against these tumor cells [1]. Given the significant success of these technologies, there is now a need for improved ways of expanding cells for administration.

The clinical efficacy of adoptive cell transfer (ACT) is largely dependent on how the antigen-specific T cells are stimulated during the priming phase [2–4] and dependent on downstream stimulation with γ chain receptor cytokines such as IL-2, IL-7, IL-15 and IL-21 [5–7]. In fact, a fine balance when dealing with artificial T cell stimulation is required in order obtain a high number of effector T cells without jeopardizing cell effectiveness. An inefficient priming may result in hyporesponsive or anergic T cells, leading to a lack of proliferation and/or loss of effector function when in contact with the respective antigen, rendering the treatment ineffective. On the contrary, excessive stimulation of the cells *in vitro* may also compromise treatment efficiency due to T cell induced cell death (AICD) [8, 9]. Therefore, novel methodologies to improve T cell priming are in high demand.

One such novel methodology is artificial antigen presentation which has emerged as a very promising concept due to its versatility both in terms of the platform used to build the system and the T cell activation method adopted. Over the past decades, several artificial antigen presenting cells (aAPCs) based on distinct systems have been developed with promising results in the *in vivo* expansion of specific T cells [10–12] with further evidence provided when applied to more advanced disease models of murine experimental autoimmune encephalomyelitis, collagen induced arthritis, non-obese diabetic animals [13] and cancer [14]. The base architecture of these systems has relied on the use of iron oxide nanoparticles [13], synthetic polymers [11], liposomes [15], magnetic beads [10, 14, 16] and cell engineering methodologies [12, 17, 18]. However, one of the greatest handicaps of these systems is the difficulty in generating, within a short period of time, large numbers of modified T cells of a defined phenotype and with a specified ratio of subsets [19]. Furthermore, in some cases, inorganic nano/microparticles, while qualified for applications such as diagnostics, imaging and photothermal therapies, have limitations related to their low solubility and toxicity concerns [20]. In contrast, biomaterials present characteristics such as biodegradability, biocompatibility, half-life and mechanical properties that can be used to shape interactions with T cells and drive immune cell responses [21]. As such, biomaterials have the potential to be used in the development of novel platforms more in tune with biological systems.

Gellan Gum (GG) is a bacterial exopolysaccharide exhibiting properties such as biocompatibility, low production costs and reproducibility over batches. GG is produced by the bacteria *Sphingomonas elodea* [22] and consists of a repeating unit of α-L-rhamnose, β-D-glucose and β-D-glucoronate in the milimolar ratios 1:2:1 [23]. GG may either exist in its native form or upon alkaline hydrolysis originate the so called low-acyl GG [22], both forms retaining the capability to crosslink into hydrogels in the presence of mono-, di- and trivalent cations [24, 25]. This polysaccharide has been proven suitable for the production of microbeads [26–28] or for the production of formulations for the sustained delivery of relevant drugs [29–35]. Due to its resemblance with the extracellular matrix glycosaminoglycan composition [36] it has gathered also important attention in the field of tissue engineering and regenerative medicine [37–40].

In this work, it has been proposed to use GG as the basis to create a multivalent T cell activator system for the controlled *in vitro* expansion of T cells. In this system, GG nano-sized particles were surface-modified with α-CD3 and α-CD28 antibodies and used to successfully prime naïve mouse T cells, generating a high number of proliferative IL-2 producing CD4^+^ T cells. Additionally, the current work shows that these cells present increased levels of T cell mediated cytotoxicity mediators Perforin 1 and Granzyme B while maintaining a low state of T cell exhaustion.

## Experimental section

### Gellan gum particle synthesis

Gellan gum nanoparticles (npGG) were produced by the double emulsion water-in-oil-in-water technique [41]. Briefly, a 1% (w/V) of gellan gum (Sigma-Aldrich, Germany) solution was prepared in deionized water, under stirring at 90 °C for 30 min. After dissolution, the solution was allowed to stabilize at 50 °C. A drop of Tween-20 (Sigma-Aldrich, Germany) was then added to the GG solution (W1) and each mL of the GG solution was emulsified in 3 mL of 0.5% of Span®80 (Sigma-Aldrich, Germany) in chlorophorm (Sigma-Aldrich, Germany)(O) while under stirring with a T18 BASIC Ultraturrax (IKA, NC, USA) for 3 min. W1/O was then added dropwise to a solution of 3.5% poly(vinyl alcohol) (PVA)(W2) (Sigma-Aldrich, Germany) in water under continuous stirring and then crosslinked by adding gradually W1/O/W2 into 5 mL of a 60% w/v solution of calcium chloride (Merck Millipore, Germany) under stirring. Chlorophorm was removed using a rotary vaccum evaporator at 37 °C (Stuart, Staffordshire, UK). The particles were recovered by centrifugation for 1 h at 13,000 × g (Eppendorf, Germany) and by passing through a series of strainers (pluriSelect, Leipzig, Germany) ranging from 10 μm down to 1 μm to limit size dispersion. In order to remove PVA, 3 additional washing steps were performed by centrifugation at 13,000 × g for 10 min with deionized water.

GG particles were then frozen [-196 °C in liquid nitrogen (N_2_)] and then freeze-dried (LyoQuest, Telstar, Spain) to obtain dried GG particles for further modification upon rehydration.

### npGG characterization

#### Chemical characterization of npGG

The presence of specific functional groups in the npGG was assessed by Fourier transform infrared spectroscopy (FTIR). To obtain the FTIR spectra of npGG, transparent potassium bromide (Sigma-Aldrich, Germany) pellets were prepared containing the samples to be analyzed. The readings were performed in an infrared spectroscope (IR Prestige-21, Shimadzu, Japan) with 4 cm^−1^ resolution and the results are presented as the average of 32 scans.

#### npGG morphology

The npGG size distribution and surface charge were analyzed by dynamic light scattering after preparing a 1 mg.mL^−1^ suspension of rehydrated GG particles in deionized water (Zetasizer Nano ZS, Malvern Instruments, UK). Acquisition was performed with the detector positioned at a scattering angle of 173°.

Particle size and morphology were analyzed with a High-Resolution Field Emission Scanning Electron Microscope with Focused Ion Beam (FIB – SEM) (AURIGA COMPACT, ZEISS, Germany). For this effect, the samples were diluted at 0.5 mg.mL^−1^ in ultrapure water followed by a further dilution of 1:20 and were dispersed onto the surface of a 400-mesh carbon-coated copper grid (Ted Pella, USA) for further observation.

#### Particle stability upon rehydration

To determine the stability of the produced GG particles over time, a 1 mg.mL^−1^ suspension of npGG was prepared in different solutions (culture medium, phosphate buffer saline (PBS), and deionized water) and filtered through a 0.8 µm filter (Millex, Millipore, France). Particle size and dispersity was evaluated over 7 days by dynamic light scattering (DLS) (Zetasizer Nano ZS, Malvern Instruments, UK). Between data acquisition times, samples were stored at room temperature.

#### Water uptake and water content quantification

The dried GG particles were rehydrated with PBS up to 7 days at 37 ºC, to determine the water uptake profile. Samples were weighed prior (W_d_) and after each time point (W_w_) and the percentage of water uptake over time was calculated based on the Eq. (1) below. The water content of the hydrogel particles was determined through of the particles in their wet state (W_w_) versus the weight in their dried state (W_d_):

$$\mathrm{Water}\;\mathrm{uptake}/\mathrm{content}\;(\%)=({\mathrm W}_{\mathrm w}-{\mathrm W}_{\mathrm d})/{\mathrm W}_{\mathrm d}\times100$$

#### Particle functionalization with antibodies

npGG in suspension were functionalized through chemical coupling by one of two methods. Briefly, 1 mg of npGG was dissolved in MES buffer at 50 mM and pH 6.5. Carboxylic groups were activated through the addition of N-(3_Dimethylaminopropyl)-N´- ethylcarbodiimide hydrochloride and N-hydroxysulfosuccinimide to achieve a final concentration of 17.3 μM and 18 μM respectively and left to stir for 1 h. The particles were then washed 3 × by centrifugation at 18,000 × g and afterwards either i) NeutrAvidin was added first and left to react over 4 h at RT under agitation after which the particles were washed again and 10 µg/20 µg of biotinylated α-CD3/CD28 antibodies (MyBioSource™, California, USA) were added or ii) 10 µg/20 µg of functional grade α-CD3/CD28 (Tonbo Biosciences™, California, USA) antibodies were added directly to the EDC/NHS activated particles and incubated overnight at 4 ºC. The following day, the produced activator-npGG were washed again 3 × by centrifugation at 18,000 × g to remove unbound antibodies and particles were suspended in adequate medium.

#### Density of NeutrAvidin

The density of NeutrAvidin protein on the particles surface was determined using the micro bicinchoninic acid assay (Pierce, Rockford, IL) in accordance with the manufacturer’s instructions. For this effect, 1 mg of GG particles was functionalized as stated above with either 0.5 mg or 1 mg of avidin by conjugation through EDC/NHS. Experimental replicates were performed for reproducibility of the chemical conjugation and elution’s resulting from washing step were stored for quantification of unbound NeutrAvidin.

#### Assessment of npGG functionalization

To evaluate the binding of functional grade antibodies to npGG’s, SDS-PAGE (Sodium, Dodecyl, Sulfate, Polyacrylamide Gel Electrophoresis) analysis was used (Sigma-Aldrich, Germany). To achieve protein separation, two separate gels were used, a 4,7% stacking gel and a 12% resolving gel. In each well, an equal amount (10 µg) of either control or modified particles were loaded after being prepared by heating at 60 ºC for 30 min prior. A calibration curve of the biotinylated antibody of 0.5 µg, 1 µg and 2 µg was performed. The direct binding of the antibody to the particles was also performed and again a calibration curve of the standard antibody was equally performed 0.5 µg, 1 µg and 2 µg. The bands in each well were observed by soaking the gel with Coomassie Blue (National Diagnostics, Atlanta, USA) followed by destaining.

### Functionality evaluation

Functionality assays were carried out to determine the *in vitro* performance of the produced activator-npGG in terms of both surface interaction with host immune cells, as well as, the capacity to trigger T cell proliferation.

#### Cell lines and culture conditions

Primary human dermal fibroblasts (hDFbs) (Gibco, UK) were obtained from Thermo Fischer and cultured in α-MEM, supplemented with 10% fetal bovine serum (FBS, Invitrogen, USA) and 1% antibiotic/antimycotic. Cells were kept in culture at 37 °C in a humidified atmosphere with 5% CO_2_.

#### Cell isolation

All procedures were approved by the Direcção Geral de Alimentação Veterinária, the Portuguese National Authority for Animal Health, and respected the national regulations and international animal welfare rules. Murine splenocytes cells were obtained from Balb/c mice. Briefly, mouse spleens were excised and sliced into small pieces. The fragments were placed onto a strainer attached to a 50 mL conical tube. The excised spleen fragments were pressed through the strainer using the plunger end of a 5 mL syringe. The cells were washed through the strainer using excess cell culture medium. Cells were collected by centrifugation at 400 × g for 5 min. The cell pellet was resuspended in 1 mL of pre-warmed (37 ºC) red blood cell lysis buffer and incubated at 37 ºC for 5 min. Murine splenocytes were then washed with excess PBS and centrifuged at 400 × g for 5 min.

Cells were cultured from hereon with RPMI 1640 medium (Sigma-Aldrich, Germany) supplemented with 10% FBS (Gibco, ThermoFisher Scientific, Paisley, UK) 1% HEPES (Gibco, ThermoFisher Scientific, Paisley, UK), 1% Sodium Pyruvate (Gibco, ThermoFisher Scientific, Paisley, UK) and 0.05 mM Mercaptoethanol (Gibco, ThermoFisher Scientific, Paisley, UK) at 37 °C in an incubator equilibrated with 5% CO_2_.

#### Metabolic activity in response to npGG

To determine the metabolic activity of cells in response to npGG, hDFbs were stimulated with different npGG concentrations (2, 20, 100, 200 µg.mL-1) for 72 h. Metabolic activity and cell proliferation were quantified after this period using CellTiter 96® AQueous One Solution Cell Proliferation Assay (Promega, USA) and Quant-iT PicoGreen dsDNA Assay Kit (Invitrogen, UK), respectively as recommended by the manufacturer. Metabolic activity data was expressed as the percentage of cell metabolic activity of the stimulated cells normalized to the non-stimulated hDFbs. The concentration of DNA present in each condition was determined against a standard curve.

#### Visualization of activator npGG-cell binding

For visualizing activator npGG-splenocyte interactions, murine splenocytes were stimulated for 5 days with varying concentrations of the developed system. At different time-points, cells were collected, washed and growth medium supernatant was aspirated and replaced with PBS at a concentration of ~ 1 × 10^6^ cells.mL^−1^. A pipette was used to gently mix and transfer 1 mL of the cell suspension into a culture plate well holding a coverslip. The culture plate was then left stationary for 30 min at room temperature to allow for the sedimentation and adhesion of cells to the coverslip. Afterwards, the PBS suspension containing remaining non-adherent cells was gently aspirated. The adhered cells were then fixed for 15 min at room temperature with ice cold methanol (Sigma-Aldrich, Germany) pipetted along the side of the well. Fixed cells were then washed with PBS and stored at 4 ºC until immunolabeling.

Primary antibody CD4 (Rabbit anti-Mouse, 1:200, NovusBiologicals, U.K.) and Alexa Fluor 488-tagged Goat anti-rabbit secondary antibody (1:500, ThermoFisher Scientific, Paisley, UK.) were used for cell visualization (Supplementary Table S1, Additional file 1). Nuclei were stained with DAPI (Invitrogen, U.S.) For particle visualization, a Donkey anti-rat AF594 secondary antibody (1:500, ThermoFisher Scientific, Paisley, UK) was used. An AXIOIMAGER Z1M (Carl Zeiss Microscopy GmbH, Jena, Germany) widefield microscope or a TCS SP8 Leica laser confocal microscope (Leica Microsystems, Wetzlar, Germany) were used for visualization of the samples.

#### Quantification of activator npGG-cell binding

To quantitively determine the percentage of binding of the activator-npGG system to T cells, freshly isolated murine splenocytes were placed in culture with varying concentrations of α-CD3 antibody-functionalized GG particles (1 μg, 10 μg, 50 μg and 100 μg) produced either by directly coupling the antibodies to the particles or through the NeutrAvidin/Biotin immobilization. Cultures proceeded over 5 days and, ultimately, cell-bound particles were labelled in a first step with a rabbit anti-rat IgG AF488 secondary antibody (1:500, ThermoFisher Scientific, Paisley, UK) followed by a wash step. T cells were then labelled with a rat anti-mouse AF647-conjugated CD4^+^ antibody (2.5 μg.mL-1, Biolegend, London, UK) and conjugation was assessed through flow cytometery.

#### T cell proliferation and activation in response to functionalized npGG

To monitor T cell expansion when stimulated with functionalized GG particles, previously isolated murine splenocytes were labeled with CFSE. In brief, 2–5 × 10^7^ cells were incubated with 2.5 µM of CFSE at 37 ºC for 10 min. Cells were then washed three times with complete medium to quench and remove unbound CFSE and were then distributed (1 × 10^6^/well) in 96 well flat bottom culture plate. As controls for T cell expansion, 3 conditions were set up: Dynabead Mouse T-Activator CD3/CD28 for T cell Expansion and Activation (Gibco, ThermoFisher Scientific, Paisley, UK), plate bound functional grade anti-CD3 antibody (eBioscience™, ThermoFisher Scientific, Paisley, UK) (2 μg.mL^−1^) or no additional stimulator.

For the coating of the 96 well plates (Cellstar M3687, Greiner Bio-One, Austria), anti-CD3 antibodies were dissolved to a concentration of 2 µg.mL^−1^ and 50 µL were added per well and left to incubate for 1 h at RT. The following day, culture plate wells were washed with complete medium to block unspecific binding of antibodies still to be added.

Different np-GG α-CD3/ α-CD28 concentrations were added (1 µg.mL^−1^, 10 µg.mL^−1^, 50 µg.mL^−1^ and 100 µg.mL^−1^) over a period of 7 days of stimulation in a ratio of 1:1. For plate bound antibody stimulation, anti-CD28 was added to a final concentration of 2 µg.mL^−1^ to each respective well. All quantifications were performed in triplicate. The proportion of dividing cells was detected by flow cytometry based on CFSE levels of gated CD4^+^ T cells [42].

#### Enzyme-linked immunosorbent assay (ELISA) IL-2 secretion assay

ELISA (Elabscience, Hubei, CN) was used to quantify the levels of IL-2 released upon stimulation with the developed activator-npGG system. Conditioned medium was collected post 7 days of stimulation with varying concentration of the npGG activator system to assess total IL-2 produced over the complete stimulation period. The procedure was carried out according to the manufacturer’s instructions and the absorbance was read at 450 nm using a Synergy HT (BioTek, Vermont, USA) microplate reader. The quantity of IL-2 protein was determined by means of a standard curve. IL-2 within the control media was undetectable during the experiments and therefore was omitted from the figures.

#### T cell exhaustion and cytotoxic capacity

Quantitative Real-Time Polymerase Chain Reaction (qPCR) was used to detect the expression of T cell exhaustion-related genes after exposure to 7 days of stimulation with either the activator-npGG system or the commercial activator beads. Primers for the tested genes and the housekeeping gene ACTβ were designed using Primer-Blast database (NCBI, Bethesda, MD, USA) (Supplementary Table S2, Additional file 1).

qPCR reactions were carried out in a MasterCycler Realplex4 (Eppendorf, Hamburg, Germany) and primer efficiency was tested out using serial dilutions of cDNA (1, 1:10, 1:100, 1:1000). For qPCR reactions, 1 μL of synthesized cDNA was used in a 20 μL reaction containing 10 μL of PerfeCTa® SYBR Green FastMix (Quanta Biosciences, Beverly, MA, USA) and forward and reverse primers at 300 nM. Reaction conditions comprised a 2 min denaturation at 95 ºC, followed by 45 cycles of 95 ºC for 10 s, a specific annealing temperature as described in Table S2 for 30 s and 72 ºC for 10 s. Products obtained from real-time PCR were subjected to melting curve analysis to check for the correct amplicon length and the absence of unspecific products. Transcript abundances were normalized to the expression of ACTB. Samples were run in triplicate in each PCR assay. Normalized expression values were calculated following the mathematical model proposed by Pfaffl using the formula: 2 ^−ΔΔCt^ (Pfaffl, 2001).

#### Analysis of helper and cytotoxic T cell subsets

To monitor T cell activation profile, splenocytes were incubated with functionalized npGG over 7 days of stimulation. Cells were then washed, permeabilized, and stained with the following antibodies: CD45-PerCP/Cy5.5, CD4-APC-H7, CD8-APC-H7 (from BD Biosciences) and Granzyme B-PE-Cy7, Perforin-APC, CD69-PE (from Biolegend) (Supplementary Table S1, Additional file 1). For intracellular staining, cells were first incubated and fixed with Reagent A-Fix and Perm™ (Thermo-scientific, Netherlands) for 15 min, and stained with the respective conjugated antibodies diluted in Reagent B- Fix and Perm™ (Thermo-scientific, Netherlands) for 30 min at RT. After washing with PBS, cells were fixed in 1% paraformaldehyde (Sigma, USA)/PBS and then analyzed on a FACSCalibur flow cytometer (BD Biosciences, Belgium) and data was treated using Cell Quest Pro version 4.0.2 (BD Biosciences, Belgium). Cells were gated according to (Supplementary Figure S1, Additional file 1).

### Statistical analysis

Graphpad software version 7.03 was used to perform statistical analysis. To determine if data sets fell in a normal distribution the Shapiro–Wilk normality test was performed. When a normal distribution was verified a one-way analysis of variance with a Tukey post-test was performed. Otherwise, data was analyzed with the Kruskal–Wallis test followed by the Dunn’s multiple comparison post-test. Results are presented as mean ± standard deviation (SD) and the significance levels between experimental groups was set for *p < 0.05, **p < 0.01, ***p < 0.001.

## Results

### GG-based particles produced by double emulsion

**Fig. 1:**
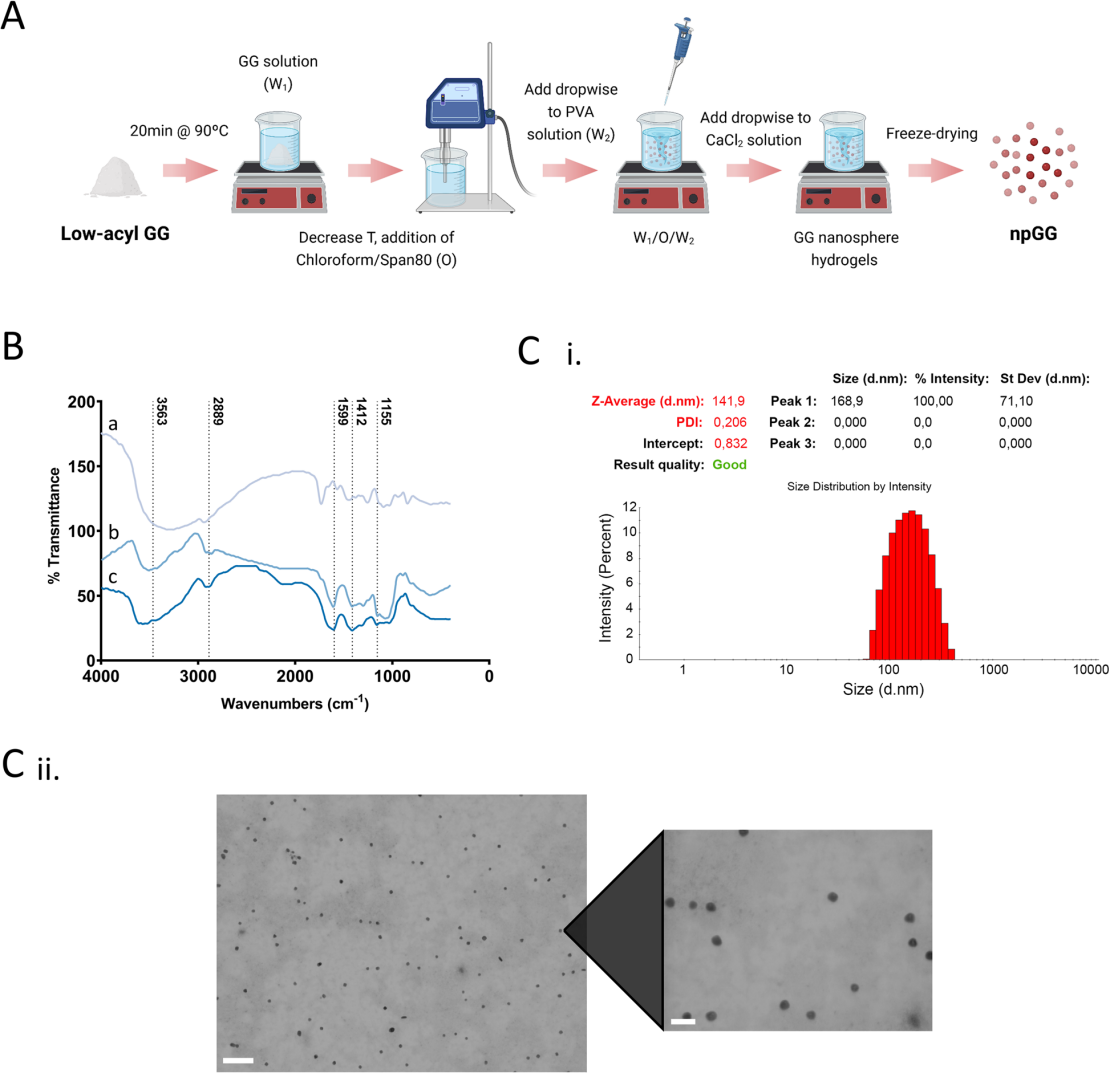
npGG production and morphological characterization. **A** Schematics of the production of npGG based on a double emulsion method. **B** FTIR spectra of (a) PVA, (b) npGG, (c) GG. **C I.** Particles size distribution of npGG according to intensity when produced by a double emulsion. **C II.** Representative STEM images of npGG. Images were obtained with an accelerating voltage of 20 kV. The scale bars represent a size of 300 nm (left) and 100 nm (right)

The core GG particles were synthesized by a double emulsion technique as represented in Fig. 1A. The particles were then subsequently evaluated in terms of both physical and chemical properties by characterizing the surface chemistry by FTIR and the particle morphology and size by DLS and FIB – SEM.

Through FTIR (Fig. 1B), the spectrum of the GG particles was compared to those of native GG and PVA in order to check for any remaining PVA in the final GG particles after the repeated washing steps. The original spectrum of GG displays characteristic transmittance bands at 3411 cm-1 indicating the stretching vibration of -OH group in the gellan gum hydrogel (Ray et al., 2010). Other peaks of free-standing GG were detected at 1051.11 cm-1 which are assigned to the C-O stretching vibration. Additional characteristic peaks at 2414 cm-1 (OH-stretching), 2924.09 cm-1 (C-H stretching), two strong peaks at 1612 cm-1 and 1413 cm-1 for asymmetric and symmetric –COO stretching and at 1028 cm-1 for C-O stretching for alky ether were equally detected. When comparing the spectrum from the GG particles with those of free standing PVA it was verified that characteristic bands, such as, the O–H stretching from the intermolecular and the intramolecular hydrogen bonds (3550 and 3200 cm − 1), the vibrational band referring to the C–H stretching from alkyl groups (2840 and 3000 cm − 1) and the peaks regarding the C = O and C–O stretching from the acetate group (1750–1735 cm − 1) are not present or if so, in a residual manner.

Regarding size analysis by DLS, a Z-average (d.nm) of 141.9 and a PdI of 0.206 was observed for the developed GG particles (Fig. 1Ci). STEM images revealed that the developed particles assumed a spherical shape in nature exhibiting a monodisperse state. Size correlating to what was obtained by DLS with variable sizes ranging from 50 nm to approximately 200 nm in diameter (Fig. 1Cii), which was expected given the double emulsion protocol used.

### GG particles retain water uptake capacity and stability over time

**Fig. 2:**
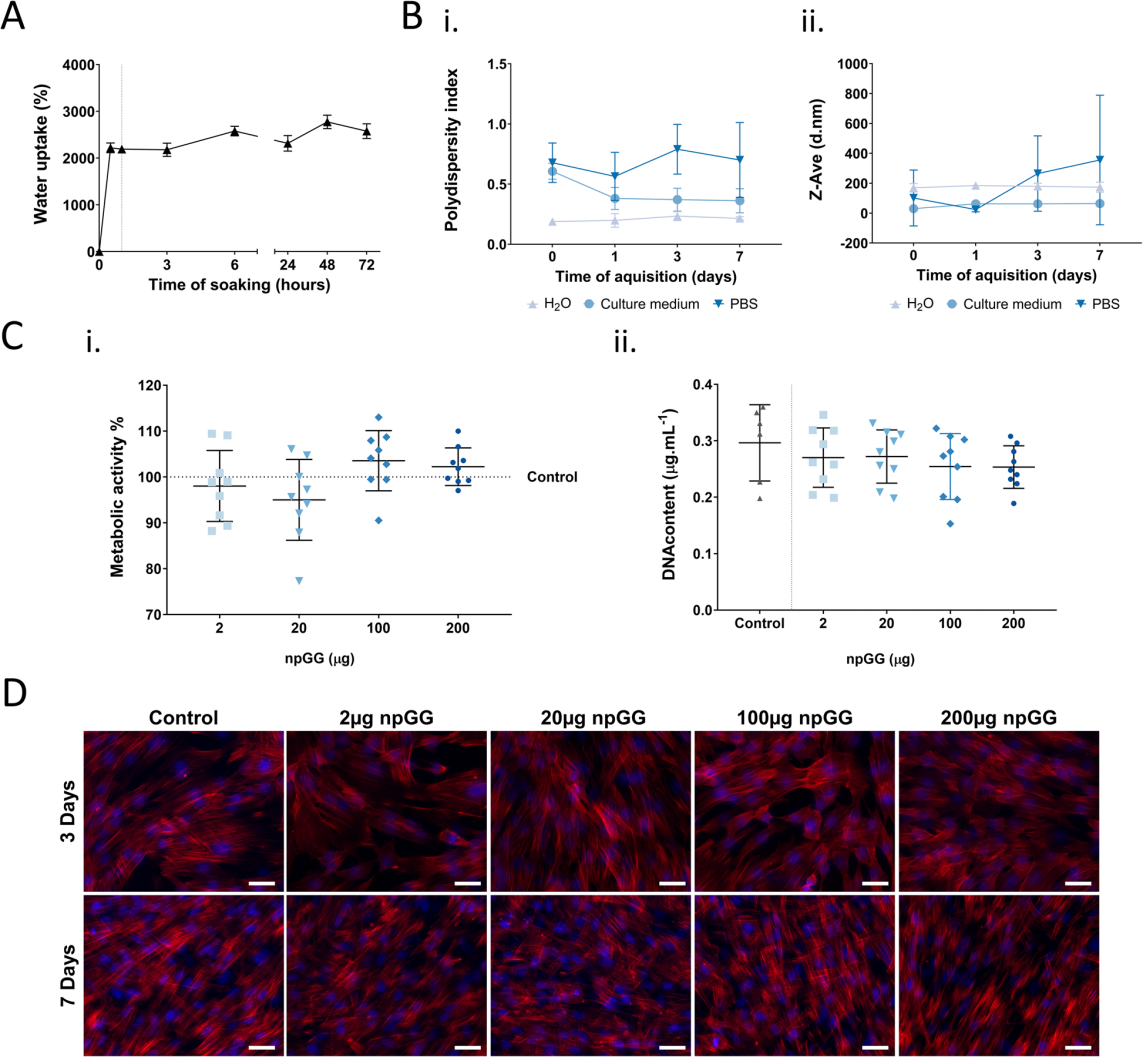
Physical and biological characterization of npGG. **A** Water uptake of dried npGG during 72 h of rehydration in PBS. **B** Effect of different solutions on npGG regarding **I.** Polydispersity index **II.** Average size distribution. **C** Biological performance of human dermal fibroblasts (hDFbs) stimulated with different npGG concentrations (2, 20, 100, 200 µg.mL^−1^) for 72 h **I.** Metabolic activity was assessed by MTS and presented as the percentage of control non-stimulated cells. **II.** Cell proliferation evaluated by DNA quantification. **D** Morphological analysis of hDFbs by rhodamine-phalloidin/DAPI after stimulation with varying concentrations of npGG (red = rhodamine and blue = DAPI). Original magnification 10 × , scale bar = 100 μm

GG solutions are known to easily crosslink in divalent cationic solutions and form hydrogels, which can withhold high contents of water. To evaluate if the developed particle system had retained this property characteristic of GG, the water uptake capacity of the particles was assessed. When the freeze-dried particles were immersed in PBS (rehydration step), a rapid weight gain of between 2700 and 3200% was observed due to water uptake (Fig. 2A).

As the produced npGG retained the feature of significant water uptake after freeze drying and are produced only by means of physical crosslinking mechanisms, the possibility that these particles become weaker in physiological conditions due to the exchange of divalent cations by monovalent ones was present. The stability of the produced particles was then determined in 3 different solutions: deionized water (dH_2_O), phosphate buffer saline (PBS) and culture medium over time by assessing the polydispersity index (Fig. 2Bi) and mean size of the particles (Fig. 2Bii). We could observe that the polydispersity was kept in an acceptable range upon solubilization of the particles in dH_2_O with a tight size variance over the 7 days period studied with an average size around 180 nm, suggesting that this system remains stable over time when in deionized liquids. Due to the high ionic content of PBS, a destabilization of the system in the form of particle agglomeration was both expected and confirmed which translated into an increased mean size and polydispersity index. Interestingly, when rehydrated in culture medium, notwithstanding the ionic content of the medium, a different behavior of the particle system was observed. Immediately after rehydration and stabilization, an increased instability of the system was verified confirmed by an increased PDI when compared to dH_2_O. However, over time, a decrease in the PDI value was observed reaching similar levels to those of dH_2_O, as well as, mean particle size averaging 64 nm.

In order to better determine the biological compatibility of the developed system, the metabolic activity of hDFbs was determined after 3 days of incubation with varying concentrations of the developed npGG. Values were normalized against dsDNA concentration and are presented as percent of the values for cells cultured in the absence of particles (Control) (Fig. 2Ci). At 3 days of incubation, metabolic activity of the hDFbs with npGG didn’t differ from the control, regardless of the npGG concentration. When looking at the dsDNA content (Fig. 2Cii) a slight decrease was present when increasing the concentration of npGG in the culture, but no statistically significant differences were found. To determine the impact on cell morphology at each end time-point, cells were fixed and labelled with Phalloidin-TRITC and DAPI. Upon visualization by fluorescent microscopy, cells presented a regular spindle shape regardless of npGG concentration (Fig. 2D).

### GG particles may be surface functionalized with biologically relevant antibodies

**Fig. 3:**
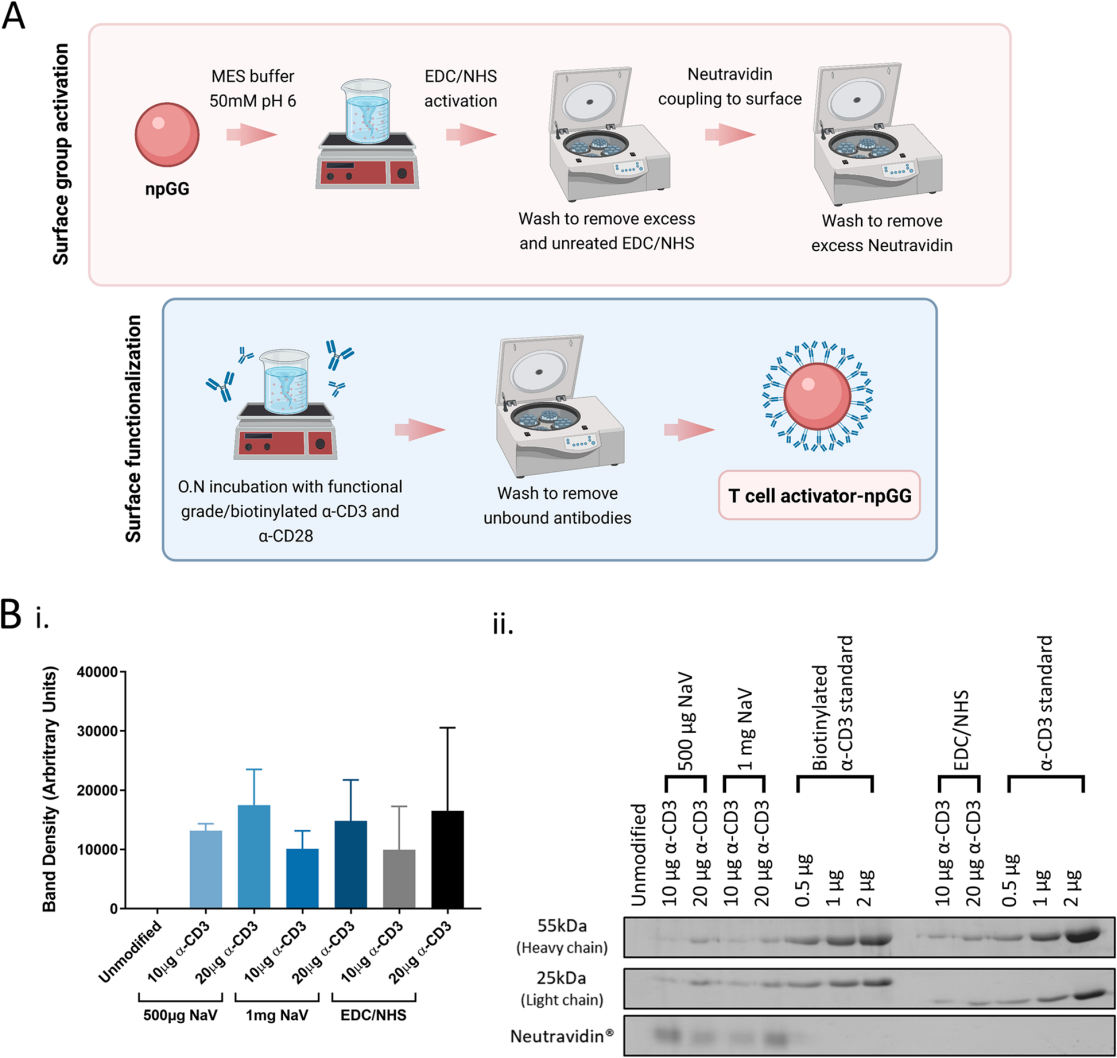
Bio-functionalization of npGG. **A** Schematic illustration of the fabrication of npGG aAPCS modified with either functional grade α-CD3 and α-CD28 or biotinylated α-CD3 and α-CD28. **B I.** Quantification by densitometric analysis of the amount of both functional grade or biotinylated antibodies bound to the surface of GG particles by NeutrAvidin (NaV) or by EDC/NHS. **C** **II.** Surface functionalization of GG particles with α-CD3 antibody and biotinylated α-CD3 were measured by SDS-PAGE

The immobilization on the surface of the GG particles of two antibodies to mimic the co-stimulatory signals critical for T cell priming, was then sought. Two different methods were used to achieve this i) direct binding of the antibodies through EDC/NHS chemistry or ii) coupling of NeutrAvidin to the surface of the particles though EDC/NHS chemistry followed by non-covalent binding of biotinylated antibodies (Fig. 3A).

To verify the stable binding of NeutrAvidin to the surface, protein content was quantified through micro bicinchoninic acid assay of both the functionalized particles and the elution fractions resultant from the washing steps. Results showed that opting for 1 mg of NeutrAvidin for the functionalization didn’t return a significant difference in bound protein when compared to 0.5 mg with protein concentrations of respectively 45 µg.mL^−1^ and 38 µg.mL^−1^ (Supplementary Figure S2, Additional file 1). Considering that that both concentrations returned a similar concentration of bound Neutravidin, a higher amount of unbound protein was expected to be found in the different elution fractions when 1 mg of NeutrAvidin was used (Supplementary Figure S2, Additional file 1). The low dispersion of values found over the different experimental replicates suggest stable binding of the protein and high reproducibility of the protocol.

Following, it was evaluated which of methods i) or ii) would lead to a more efficient binding of the desired antibodies. For this effect, both systems, either functionalized with NeutrAvidin or not, were reacted with two amounts of anti-CD3 antibody, 10 and 20 µg and were subsequently blotted by SDS-PAGE (Fig. 3B). While a trend for a higher amount of bound antibody was observed when using 20 µg, this change wasn’t statistically significant. All subsequent particles for this study were then functionalized with either 10 µg/20 µg of antibody directly bound to the particle or 10 µg/20 µg of biotinylated antibody bound to particles previously incubated with 0.5 mg of NeutrAvidin in order to determine the effects of the antibody concentration on the *in vitro* biological performance of the system.

### Surface interaction between CD4^+^ T cells and activator-npGG

**Fig. 4:**
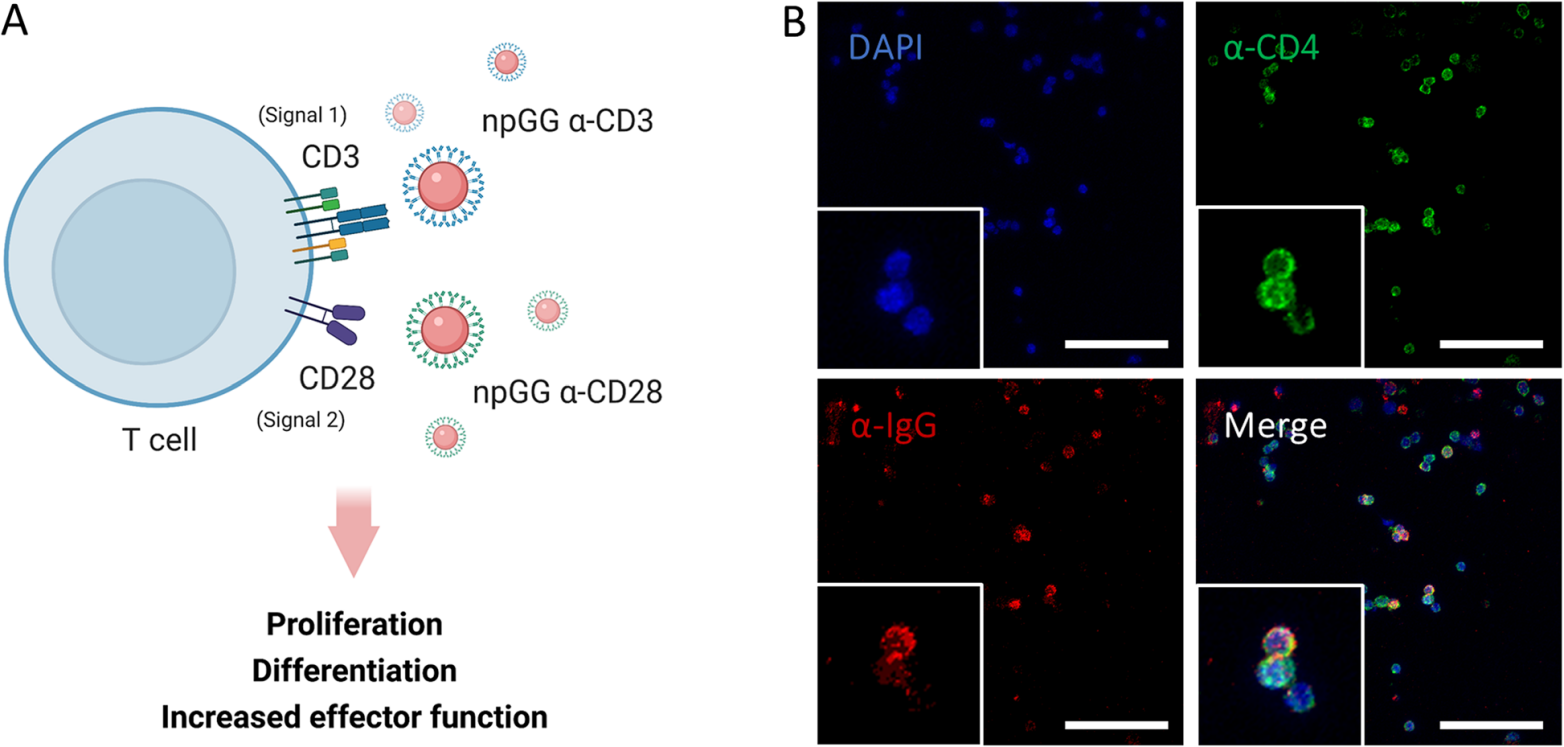
Characterization of the interaction of npGG aAPCs with murine T cells. **A** npGG aAPCs interaction and T cell interaction schematics. **B** Confocal images of 100 μg of npGG-α-CD3 surface-bound to CD4^+^ T cells after 3 days of culture. Green fluorescence shows CD4^+^ T cells, while red represents npGG surface bound α-CD3, scale bar = 50 μm

From a biological point of view, the key characteristic of an aAPC is its capability to specifically stimulate T cells of interest to avoid off-target inflammation and autoimmunity events. Taking this into account, the ability of the developed activator-npGG to interact with the surface of CD4^+^ T cells and bind to the CD3 receptor was evaluated (Fig. 4A). By labelling both CD4^+^ and or activator-npGG, it was possible to check for double positive cells indicating particles bound to the desired T cells.

Quantitative analysis of the binding of activator-npGG to the surface of CD4^+^ regulatory T cells was performed by flow cytometry over 2 distinct time-points, 1 and 5 days of incubation (Table1). After 24 h, a dose-dependent binding of particles to cells was verified, with increasing particle concentration leading to increased binding. A significant amount of particle-CD4^+^ T cell binding was detected in doses of both 50 µg and 100 µg when modified with 20 µg of antibody. Additionally, activator-npGG prepared by direct linkage of antibodies to the particle surface resulted in an overall lower significance (*p* < 0.01) in the number of cell bound particles when compared to the NeutrAvidin-biotin system (*p* < 0.001). Upon looking at cell interaction at day 5, a similar trend to that of the day 1 time-point was observed in which a significantly higher binding of npGG-α-CD3 to CD4^+^ T cells was observed when higher doses of the 20 µg α-CD3 were used in the NeutrAvidin-based particles.

**Table 1:** Surface interaction between npGG aAPCs and murine CD4^+^ T cells

Experimental conditions	µg	%	
		**Day 1**	**Day 5**
**npGG**	1	1.68 (0.36)	1.1 (1.13)
	10	2.26 (0.71)	1.63 (1.39)
	50	2.4 (1.09)	1.65 (0.96)
	100	1.8 (0.53)	1.9 (1.50)
**npGG 10 µg α-CD3**	1	3.71 (2.09)	8.4 (10.03)
	10	10.65 (9.88)	8.57 (6.53)
	50	22.82 (16.67)	19.53 (18.05)
	100	30.79 (2.09)	19.53 (18.05)
**npGG-NaV 10 µg α-CD3**	1	3.68 (2.16)	4.98 (3.52)
	10	12.3 (9.21)	11.57 (7.86)
	50	37.57 (33.04)	19.8 (15.73)
	100	39.21 (31.75)	33.34 (23.23)
**npGG 20 µg α-CD3**	1	6.65 (1.91)	6.8 (5.24)
	10	22.83 (8.35)	17.23 (4.46)
	50	38.48 (15.79)	40.4 (30.74)
	100	70.6 (0.60) **	44.1 (18.29)
**npGG-NaV 20 µg α-CD3**	1	9.7 (1.67)	8.55 (3.61)
	10	35.5 (6.79)	28.13 (5.54)
	50	51.45 (17.75) ***	53.65 (8.33) **
	100	61.53 (8.04) ***	61.1 (8.08) ***

Flow cytometry data of murine splenocytes incubated with varying concentrations of npGG aAPCs over 1 and 5 days in the percentage of CD4^+^ T cells positive for nanoparticles. CD4^+^ T cells were labeled with AF647 while npGG-α-CD3 were labelled with AF488 anti-rat IgG. Double positive labeling was translated in the % of CD4^+^ T cells with surface bound npGG-α-CD3. Quantitative results are expressed as the mean ± standard deviation where *n* = 3, * *p* < 0.05, ** *p* < 0.01, *** *p* < 0.001, **** *p* < 0.0001, one-way ANOVA with Kruskal–Wallis multiple comparison post-test

When correlating the quantative data with that of immunolabelling (Fig. 4B) it was possible to verify a homogenous distribution of the NeutrAvidin based activator-npGG throughout the surface of CD4^+^ T cells when 100 µg of the 20 µg α-CD3 formulation was used.

### Activator-npGG induced a dose dependent priming of CD4^+^ T cells

**Fig. 5:**
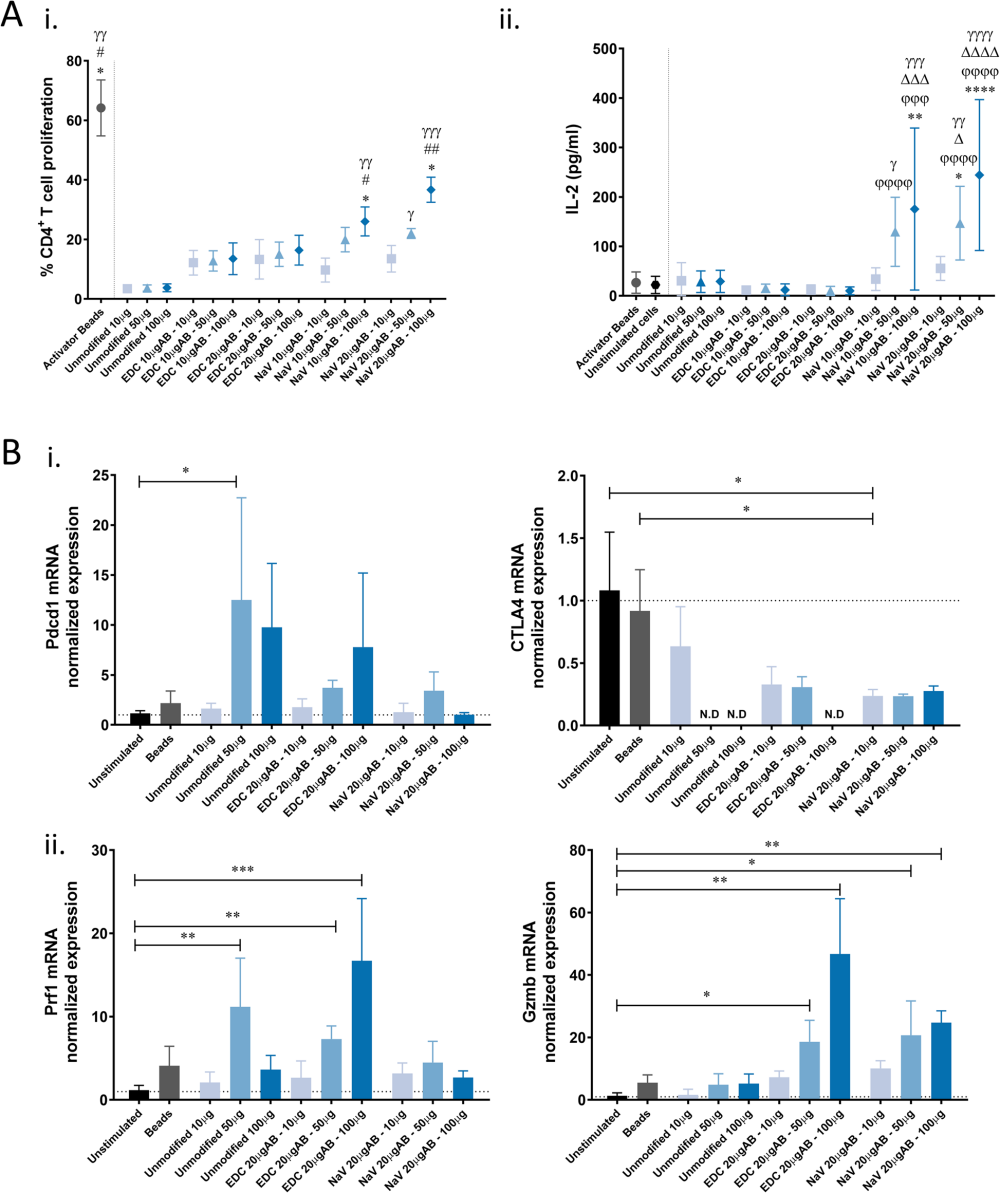
α-CD3/α-CD28 npGG can stimulate T cells activation **A** Activation profile of murine splenocytes when stimulated with varying concentrations of npGG aAPCs. **I.** Measurement of CD4^+^ T-cell proliferation in vivo by CFSE dilution. Murine splenocytes were labelled CFSE and then stimulated with both α-CD3 and α-CD28 npGG in a ratio of 1:1 for a period of 7 days. After this period cells were labelled and gated for anti-CD4 and analyzed on a BD FACSAria™ III. Results are presented as the percentage of cells in the final population that have divided. Data are presented as means ± standard error. *, #,γ *p* < 0.05; **, ##, γγ *p* < 0.01; ***, ###,γγγ *p* < 0.001 relatively to unmodified particles at 10 μg, 50 μg or 100 μg respectively. **II.** IL-2 production by murine splenocytes when stimulated over 7 days with both anti-CD3 and anti-CD28 GG particles in a ratio of 1:1. Control (CTRL) corresponds to the conditions in which unmodified particles were used to stimulate the cell cultures. The IL-2 contents of the control media was undetectable. Data are presented as means ± standard error. *, ϕ, Δ, γ *p* < 0.05; **, ϕ ϕ, Δ Δ, γγ *p* < 0.01; ***, ϕ ϕ ϕ, Δ Δ Δ, γγγ *p* < 0.001; ****, ϕ ϕ ϕ ϕ, Δ Δ Δ Δ, γγγγ *p* < 0.0001. * was used when pairwise comparisons are performed relative to unmodified particles at 10 μg, 50 μg or 100 μg respectively. ϕ was used when comparisons are performed relative to activator beads, Δ relative to unstimulated cells and γ relatively to EDC at comparative 10 μg or 20 μg of antibody at 10 μg, 50 μg or 100 μg respectively. **B** qPCR mRNA fold change of **(i)** immune checkpoint genes *PDCD1* and *CTLA4* **(ii)** cytotoxic genes *PRF1* and *GZMB* in murine splenocytes stimulated with activator-npGG over 7 days. Quantitative results are expressed as the mean ± standard deviation where *n* = 3, * *p* < 0.05, ** *p* < 0.01, *** *p* < 0.001, **** *p* < 0.0001. One-way ANOVA with Kruskal–Wallis multiple comparison post-test were used

Upon verification that the developed activator-npGG could efficiently bind to the surface of CD4^+^ regulatory T cells, it was hypothesized that by co-stimulating murine splenocytes with both npGG-αCD3 and npGG-αCD28 particles it would be possible to efficiently prime T cells to expand. To verify this, freshly isolated BALB/c splenocytes labelled with CFSE were co-stimulated with varying concentrations of both npGG-αCD3 and npGG-αCD28 particles in a 1:1 ratio. CFSE dilution was analyzed by flow cytometry after 7 days to determine the percentage of proliferating cells. Effective stimulation of CD4^+^ T cells only occurred at higher doses of 50 µg and 100 µg of both npGG-αCD3 and npGG-αCD28 particles (Fig. 5Ai). Stimulation happened regardless of the activator-npGG being produced by direct coupling of the antibodies or through NeutrAvidin, however, statistically significant differences were only observed when NeutrAvidin-Biotin was used to bind the antibodies. Some activation in the control condition consisting of non-functionalized particles was also evident, which may be possibly explained due to the use of a heterogenous population of cells in which host APCs may be presenting particles to T cells. The ELISA results (Fig. 5Aii) demonstrated that IL-2 levels for splenocytes stimulated with npGG-αCD3 and npGG-αCD28 were much higher when treated with doses of both 50 µg and 100 μg of NeutrAvidin/antibody-containing particles. Moreover, these particles showed significantly higher levels of released IL-2 when compared to commercial activator beads when used as recommended by the manufacturer.

### Activator-npGG present diminished levels of T cell exhaustion and enhanced cytotoxicity

The potential of the npGG-αCD3 and npGG-αCD28 particles to induce a cytotoxic profile in T cells while avoiding their early exhaustion and senescence was determined using qPCR after 7 days of murine splenocyte stimulation. The mRNA expression levels for *PDCD1, CTLA-4, PRF1* and *GZMB* were studied. Results showed a significant increase (*p* < 0.05) of *PDCD1* expression (Fig. 5Bi) in the unmodified particles group at doses of 50 μg. However, no significant differences in expression were found when comparing stimulated splenocytes, unstimulated splenocytes and the commercial activator beads. Interestingly, a significant (*p* < 0.05) downregulation of cytotoxic T-lymphocyte antigen 4 (*CTLA4*) expression was observed in the NeutrAvidin-Biotin activator-npGG system when bound to 20 μg of antibody when comparing with both unstimulated cells and the commercial activator beads group (Fig. 5Bi) suggesting decreased repression in T cell function and therefore a more sustained immune response upon activation with these particles. Concerning whether there was a switch to a cytotoxic T cell profile, a significant (*p* < 0.01) increase in the expression of *PRF1* (Fig. 5Bii) was seen in a dose dependent manner when 20 μg of antibody was conjugated to the developed system via EDC but not via NeutrAvidin-Biotin. On the other hand, mRNA levels of *GZMB* (Fig. 5Bii) were significantly increased for both activator-npGG systems when stimulating with combined doses of 50 μg and 100 μg of both npGG-αCD3 and npGG-αCD28 when compared to unstimulated cells indicative of an increased cytotoxic profile of these cells.

### Activator-npGG favor T cell activation and helper and cytotoxic subsets

**Fig. 6:**
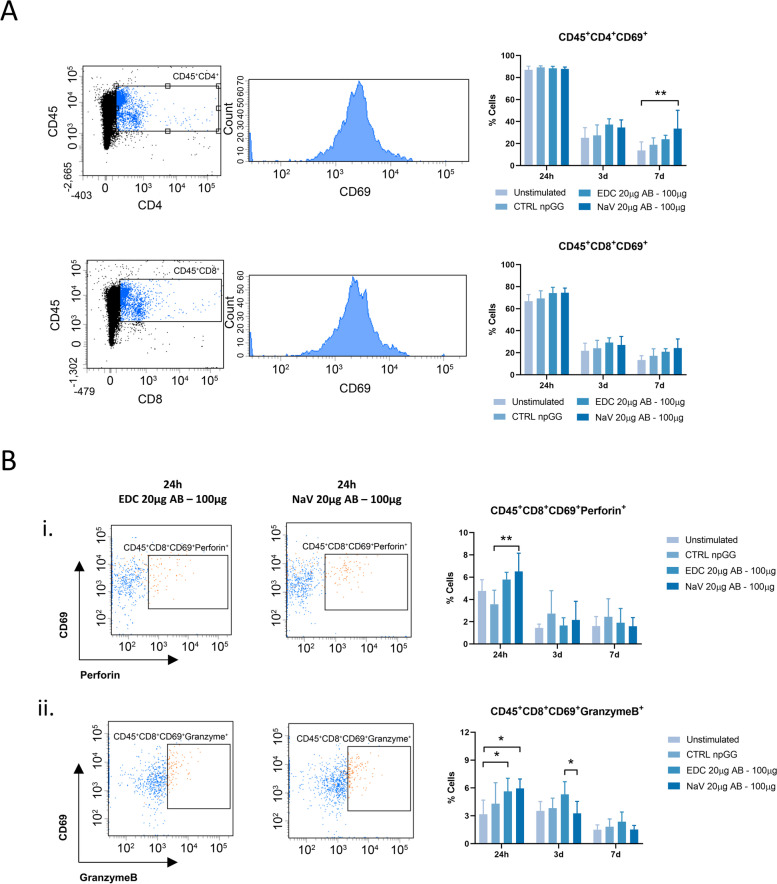
Cytotoxic T cell responses of murine splenocytes stimulated with npGG aAPCs **A** T cell activation profile of freshly isolated splenocytes stimulated with activator-npGG over 24 h, 3 d and 7 d. Activated cells were gated as CD45^+^CD4^+^CD69^+^ and CD45^+^CD8^+^CD69^+^ T cells on a BD FACSAria™ III. Summarized data is shown as bar graphs. **B** Flow cytometric plots of intracellular staining and frequencies of expression of Perforin **(i)** and Granzyme B **(ii)** in CD45^+^CD8^+^CD69^+^ T cells are shown. Quantitative results are expressed as the mean ± standard deviation where *n* = 3, * *p* < 0.05, ** *p* < 0.01, *** *p* < 0.001, **** *p* < 0.0001, two-way ANOVA with Tukey multiple comparison post-test

To explore the degree of T cell activation resulting from the stimulation of murine splenocytes with the activator-npGG functionalized with either EDC/NHS or NeutrAvidin/Biotin, flow-cytometric assessment was performed for the following surface markers: CD69, which is an early T cell activation marker; CD4^+^, which is a T helper cell marker (CD4^+^ T cells); CD8, which is a cytotoxic T cell marker (CD8^+^ T cells); and CD45, which is pan-leukocyte marker. Intracellular cytokine expression levels Perforin and GranzymeB (GrzB) were also examined. The percentage of CD69^+^ cells among CD4^+^ and CD8^+^ T cells were comparable at 24 h of stimulation (Fig. 6A). Interestingly, a differential production of Perforin and GrzB was observed (Fig. 6B). Perforin and granzymes work synergistically to induce apoptosis in target cells recognized by cytotoxic T lymphocytes. A significant increase in the expression of intracellular perforin in cells stimulated with NeutrAvidin functionalized activator np-GG was observed, while no significant differences were observed for GrzB at 24 h of stimulation. At 3 days of stimulation, despite CD69 being an early activation marker, a trend towards a greater activation of CD4^+^ and CD8^+^ T cells in both modified particles versus the control was observed (Fig. 6A). Intracellular levels of GrzB showed to be increased when T cells were stimulated with activator-npGG produced via EDC/NHS, when compared to the remaining conditions (Fig. 6B). After 7 days of stimulation, changes among the CD69^+^ population of CD4^+^ and CD8^+^ T cells were clear (Fig. 6A). A significant increase (*p* < 0.01) in activation was evident in the NeutrAvidin functionalized activator-npGG when compared to the remaining conditions. Regarding the expression of GrzB and perforin (Fig. 6B) no statistically significant changes were verified at this timepoint although a trend similar to the one verified for the 3 day time point was observed.

## Discussion

Immunotherapy has recently been in the spotlight for the treatment of various pathologies varying from cancer to auto-immune diseases. Over the years, several different systems ranging from nanoparticles, nanogels, inorganic nanoparticles, cell derived nanoparticles to microparticles, microcapsules, as well as, both living and artificial cells have been applied to develop novel immunotherapeutics. All of these possess unique characteristics with proven efficacies in several fields. A fine tailoring of these systems in order to attain specific interactions with host cells is required in order to achieve an effective and persistent T cell priming without over-activation. An imbalance within these stimulatory mechanisms regularly leads to responses of an immunosuppressive nature [8, 9].

The present study demonstrates the potential of the natural origin polymer GG as a source for the development of nanogels as fully functional T cell activator systems capable of i) generating large numbers of CD4^+^ regulatory T cells, ii) preventing T cell exhaustion when compared to other commercial artificial systems, iii) being highly tailorable to carry any desired molecule of choice and iv) present the same high capacity of water uptake of previously reported spongy-like hydrogels which might be of interest for downstream uptake of biologically relevant molecules for controlled release. Furthermore, nanogels have been shown before to present improved circulation, biodistribution and adjuvancy [43]. Other nanoparticle-based systems have also been described for immunomodulation and for immunotherapeutic approaches. Some of these systems, recently reviewed by Park et al. [44] and Shin et al. [45], have been used for antigen and adjuvant delivery as well as for the modulation of the immune-suppressive tumor microenvironment. The efficacy of nanoparticles in efficiently carrying out these functions is mainly dependent on nanoparticles properties such as particle size, surface charge, shape, and hydrophobicity [44]. The developed npGG particles within this work presented a size of approximately 140 nm and a round morphology which has been shown to condition nanoparticles to ECM entrapment and restrict their circulation to lymph nodes [46], particularly suiting this current application since tumor-specific CD8 T cells are activated in the tumor-draining lymph nodes.

Having developed the above-mentioned core system, understanding its stability was of importance to determine how to store it over time while maintaining a homogenous distribution of the particles in solution. PBS was shown to clearly interact with the particles over time, which may be explained by the ionic exchange between the solvent and the particles, leading to agglomeration and an increase of the crosslinking ratio due to the introduction of new ions in the polymeric chains of the particles [38, 47]. Interestingly, when rehydrated in culture medium, notwithstanding its ionic content, a different behavior of the particle system was verified. Immediately after rehydration, increased instability of the system was observed and confirmed by increased PDI when compared to dH_2_O. However, eventually, a decrease in the PDI value was observed which can be explained by the formation of a protein corona around the particles blocking further ionic exchange and therefore stabilizing their behavior. Deionized or slightly ionic solutions seem therefore the best alternatives to both swell the particles prior to use or for storage.

While relatable in terms of application with other acellular nanoparticles systems, the activator-npGG introduce a new variable which is the capability of high water uptake after freeze-drying, characteristic of GG spongy-like hydrogels [36]. This is an interesting feature as it may be used downstream for the uptake of biologically relevant molecules and their controlled release *in situ*, such as in the case of IL-1β which has been shown to favor the expansion of both naïve and memory CD4^+^ T cells when challenged with an antigen [48]. In addition to IL-1β, other strategies have been proposed, namely the delivery of oxygen to attenuate the hypoxic TME or the delivery of alkaline buffers to control TME pH, or even collagenases and hyaluronidase to allow for tumor ECM penetration of other therapeutic agents [45]. The delivery of adjuvants such as 3-O-desacyl-4′-monophosphoryl lipid A (MPLA), lipopolysaccharide (LPS), CpG oligodeoxynucleotides (ODNs), polyinosinic:polycytidylic acid (poly I:C), and agonists of the stimulator of IFN genes (STING) have also been used to increase immunogenicity and promote the anti-cancer immune responses [44].

To carry out the stimulation of relevant T cell subsets, a stable interaction between the particle system and the targeted cell population must be assured. As stated above, as T cell activation requires a balanced priming of the cells, the assurance that the co-stimulatory molecules are delivered equally is a requirement. First, the double emulsion protocol developed aimed at achieving an average particle size in the nanoscale (50–200 nm range). However, the recommended particle size to induce T cell priming has been widely debated. Sizes can go from the micron scale [49–51] to better mimic APCs true size, down to the nanoscale [52–54] in order to achieve a better distribution of particles to cells. The latter was then chosen, expecting to achieve a better distribution of the activator-npGG over the cell membrane of the targeted T cells due to a greater surface area-to-volume ratio [55]. Then, efficient binding of the biological relevant molecules to the particles must be achieved. One of the most recent methods to be proposed for this purpose is click chemistry as reviewed by Yi et al. for nanoparticle functionalization [56]. This method has produced promising results as seen by the work of Zhang et al. where versatile aAPCs were developed from biomimetic magnetosomes decorated with stimulatory signals by using copper-free click chemistry [57]. Other reports have made use of click chemistry for the modification of nano-gels for the delivery of doxorubicin while trying to overcome multi-drug resistance [58]. In the present work, two different approaches have been tested to tackle this issue. First, direct coupling of functional grade antibodies, knowing this could result in a random orientation of the antibodies on the surface of the particles resulting from the unspecific binding of the -COOH groups from GG to the -NH of the antibodies. This could in turn lead to the blockade of the light chains of the antibody causing loss of functionality. To overcome this, a second approach using NeutrAvidin as an anchoring system for biotinylated antibodies was employed, as this method of binding would result in effective orientation of the microdomains, preserving their capability of interacting with their specific binding cites (CD3 and CD28 on the surface of T cells). It was found that by using NeutrAvidin, a more stable interaction between the npGG and T cells over time was favored, as shown by flow cytometry, therefore increasing the possibility of immune synapse formation, an essential prerequisite for T cell activation. In alternative to both of the proposed methods, click chemistry could be used by copper-free strategies based on the reaction of a diarylcyclooctyne moiety (DBCO, or ADIBO) with an azide-labeled antibody. This would allow to better control the reacting moieties in a site specific manner, which is not attainable by EDC-NHS.

To determine the functionality of the npGG, it was explored whether upon interaction the system would be capable of functionally inducing T cells to undergo serial activation, proliferation, and differentiation. Considering fluorescence dilution of CFSE-labeled T cells analyzed by flow cytometry, cell proliferation directly correlated with binding results between T cells and activator-npGG’s, in which a higher interaction between particles and CD4^+^ T cells translated into a higher percentage of proliferating T cells. Moreover, a single administration of the developed NeutrAvidin-based activator-npGG triggered increased proliferation of this T cell subset when applied at higher doses of 50 μg and 100 μg, albeit the percentage of proliferating cells being lower than commercial T cell activator microbeads.

T cell function and proliferation are deeply associated to effector molecules such as IL-2. This interleukin is critical for the development, regulation, proliferation and maintenance of regulatory T cells (Tregs) in peripheral tissues [59]. It is implicated in the effector activities of CD8^+^ T cells by inducing the expression of IFN-γ [60], TNF-α, and lymphotoxin α [61], by stimulating expression of the cytolytic effector molecules granzyme B, perforin, and by promoting effective target cell killing [62]. However, when delivered systemically, high doses of IL-2 or other cytokines often lead to high toxicity such as the cytokine release syndrome as well as low efficiency in terms of function [63, 64]. Several methods described until now usually tether relevant biomolecules to the surface of the particles resulting in delivery but not steady release of these molecules. Others have reported encapsulation during particle synthesis which many times implies exposing these molecules to aggressive chemical agents used during the processing. The NeutrAvidin-based activator-npGG herein presented, brings the possibility of foregoing the requirement of IL-2 supplementation for the expansion of T cell subsets as shown by the production of significantly higher levels of IL-2 over the period of 7 days, even when compared to the tested commercial activator micro-beads.

T cells are known to enter into a state of “exhaustion” when chronically exposed to an antigen-rich environment, therefore losing their ability to kill cancer cells or virus infected cells [65]. Exhaustion is a state that affects both long lived central memory and short-lived effector memory T cells, with debilitating consequences to immune functions. Cells undergoing exhaustion develop changes in cytokine synthesis, such as loss of secretion of IL2, IFNγ and TNFα [66]. T cell exhaustion is also characterized by an upregulation in the expression of inhibitory molecules such as programmed death receptor 1 (PD1), CTLA4, T cell immunoglobulin and mucin domain-containing molecule 3 (TIM3), lymphocyte activation gene 3 (LAG3) or CD244 (2B4) [67]. This phenomenon could explain the lower levels of secreted IL-2 for the commercial activator system despite the higher levels of proliferation registered. Hence, the expression of those inhibitory molecules was analyzed in cells stimulated with both the commercial and the developed activator systems. It was possible to demonstrate that the npGG system coupled to α-CD3 and α-CD28 antibodies was not only capable of successfully inducing T cell activation but also triggered a low expression of T cell exhaustion markers CTLA-4 and PD-1. This suggests that the developed system may stimulate cells to be active for a longer period of time when compared to commercial activator beads which triggered a high expression of CTLA-4 in cells after a period of 7 days of stimulation. While maintaining a low degree of T cell exhaustion is crucial for applications such as cancer therapy, the induction of a cytotoxic phenotype is essential. In tumor surveillance, the killing of tumor cells has been associated to the release of a variety of cytolytic enzymes [68]. A subpopulation of CD4^+^ T cells that harbors cytolytic activity through perforin/granzyme and Fas-FasL pathways has been reported [69]. Granzymes A and B are among the most abundant cytolytic enzymes, capable of inducing target cell apoptosis through caspase-dependent and -independent pathways [70]. However, for these cytolytic enzymes to be effective they must be released together with pore-forming protein perforin to form cytotoxic granules. Upon T cell recognition, the release of cytotoxic granules in the immunological synapse induces apoptosis of the target cell. These findings show that the developed activator-npGG system triggered increased mRNA expression of these factors when compared to both unstimulated cells and commercial activator beads. This may be advantageous in terms of clinical application, as cells expanded in vitro for reinfusion will present a lower susceptibility to early exhaustion and, while capable of producing increased levels of IL-2, they will also present an increased granule-mediated cytotoxicity.

Taking into consideration the aforementioned results in terms of T cell proliferation, the differences observed between the different antibody tethering strategies were further explored. While the direct effects of free Streptavidin/Biotin in immune cells have been studied [71, 72], the differences that these conjugation methods may have on a final aAPC system have never been considered in the context of helper and cytotoxic T cell activation. Given the differential expression of Prf1 and GrzB observed in the different experimental conditions, it was hypothesized whether there was a specific effect on the fate of the developed immune response in consequence of the different antibody tethering strategy used. Hence, the activation of both CD4^+^ helper or CD8^+^ cytotoxic T cells and consequently their production of perforin and GrzB for both tethering strategies was evaluated by flow cytometry. Although CD69 is considered as an early activation marker in T cells [73], within 24 h of stimulation no detectable differences were observed among the conditions analyzed in within this work, with high percentages of CD69^+^ cells for all conditions. This result comes into agreement with prior reports which show an up-regulation of CD69 in freshly isolated CD4^+^and CD8^+^ peripheral blood lymphocytes [74]. This may be associated to a hyperresponsive state of the cells due to the isolation procedure. At day 7 of stimulation, while not statistically significant, a clear trend where particles functionalized via NeutrAvidin conjugation presented the highest levels of activated CD4^+^ and CD8^+^ T cells was observed. The fact that activation was detected at such a late time point could be explained by the fact that the splenocytes undergoing activation with the current system exhibited a more prolonged kinetics of induction, confirmed by a high percentage of proliferating CD4^+^ T cells at this time point, as well as by decreased levels of the T cell exhaustion marker CTLA4. This comes into agreement with previous reports where it was shown that CTLA-4 controls the threshold of productive TCR signaling [75]. Interestingly, the production of intracellular cytokines was noticeably higher within the first two time points of 24 h and 3 days. Real-time PCR results show that promoting the coupling of the antibodies via EDC/NHS chemistry led to higher levels of expression of the *GZMB* genes which comes into agreement with what was observed via flow cytometry. On the other hand, while these results show a higher gene expression of *PRF1* resultant from the stimulation with the EDC/NHS activator-npGG system, intracellular perforin is observed in higher amounts in NeutrAvidin as early as 24 h after stimulation suggesting that the latter system may be more effective at inducing a cytotoxic T cell phenotype while capable of equally inducing cell proliferation.

## Conclusions

In summary, a novel type of functional T cell activator system was successfully fabricated based on a nanoscale type of hydrogel. Considering the initial design, the surface of npGG was engineered with either α-CD3 or α-CD28 antibodies and used simultaneously to induce co-stimulation of T cells. While retaining major features of hydrogels such as bio-friendly environment and high water uptake, which could be used for small molecule controlled release, the created npGG displayed highly tailorable properties for its use as a platform for the production of aAPC systems for immunotherapy strategies.

When stimulating naïve murine splenocytes, the activator-npGG system showed favorable CD4^+^ T cell activation function, triggering significantly enhanced T cell proliferation and increased secretion of cytotoxic factors, while evading T cell exhaustion.

All these results indicate that this activator-npGG system is a promising platform for T cell activation and re-education applications such as the expansion of aspecific complete T cell populations of cancer patients.

## Supplementary Information

**Additional file 1. **Supplementary Figures of Manuscript. Supplementary tables and figures required for the better comprehension of the manuscript.

## Data Availability

All data needed to evaluate the conclusions in the paper are present in the paper and/or the Supplementary Materials. Additional data related to this paper may be requested from the authors.
