# Supplementary material for: Highly tailorable gellan gum nanoparticles as a platform for the development of T cell activator systems
Source: Biomater Res. 2022 Sep 30;26:48. doi: 10.1186/s40824-022-00297-z (PMC9523970; doi:10.1186/s40824-022-00297-z)
Supplement: Supplementary file 1 — Additional file 1. Supplementary Figures of Manuscript. Supplementary tables and figures required for the better comprehension of the manuscript. [file 40824_2022_297_MOESM1_ESM.docx]

**Table S1.** Primary and secondary antibodies used for used for immunolabelling and particle functionalization.

|  |  | Cat# | Host | Target |
| --- | --- | --- | --- | --- |
| In Vivo Ready Anti-Mouse CD3 (17A2) | Tonbo Biosciences™ | 40-0032-U500 | Rat | Mouse |
| Anti-Mouse CD3 Monoclonal Antibody (BIOT conjugated) clone 17A2; Rat IgG2b,kappa | MyBioSource | MBS2534030 | Rat | Mouse |
| In Vivo Ready Anti-Mouse CD28 (37.51) | Tonbo Biosciences™ | 40-0281-U500 | Hamster | Mouse |
| Hamster Anti-mouse CD28-BIOT, clone:37.51 | MyBioSource | MBS673299 | Hamster | Mouse |
| APC anti-mouse Perforin Antibody | Biolegend | 154304 | Rat | Mouse |
| PE/Cyanine7 anti-human/mouse Granzyme B Recombinant Antibody | Biolegend | 396410 | Rat | Mouse/Human |
| PE anti-mouse CD69 Antibody | Biolegend | 104507 | Armenian Hamster | Mouse |
| APC-H7 Rat anti-Mouse CD4 | BD Pharmingen™ | 560181 | Rat | Mouse |
| APC-H7 Rat anti-Mouse CD8a | BD Pharmingen™ | 560182 | Rat | Mouse |
| PerCP-Cy 5.5 Rat Anti-Mouse CD45 Clone 30-F11 (RUO) | BD Pharmingen™ | 550994 | Rat | Mouse |
| Alexa Fluor 647 anti-mouse CD4 Antibody | Biolegend | 100424 | Rat | Mouse |
| PE Hamster Anti-Mouse CD3e | BD Pharmingen™ | 553064 | Armenian Hamster | Mouse |
| CD4 Antibody (GK1.5) [FITC] | Novus Biologicals | NBP2-26584 | Rat | Mouse |
| CD4 Antibody (GK1.5) [PE] | Novus Biologicals | NBP2-26586 | Rat | Mouse |
| Alexa Fluor 594 Donkey anti-Rat IgG (H+L) | Thermo Fisher Scientific Inc. | A-21209 | Donkey | Rat |
| CD4 Antibody | Novus Biologicals | NBP1-19371 | Rabbit | Mouse |

**Table S2.** Sequence of primers used for RT-PCR studies.

| **Gene and accession number** | **Sequence (5'-3')** | **Amplicon size (bp)** | **Annealing temperature (ºC)** |
| --- | --- | --- | --- |
| Mouse Actb  NM_007393.5 | GATCAAGATCATTGCTCCTCCTG | 183 | 60 |
|  | AGGGTGTAAAACGCAGCTCA |  |  |
| Mouse Actg1  NM_009609.3 | CTTACACTGCGCTTCTTGCC | 78 | 60 |
|  | GTGCGGCGATTTCTTCTTCC |  |  |
| Mouse Pdcd1  NM_005018.2 | AACCCTGGTGGTTGGTGTC | 107 | 55.5 |
|  | TGGCTCCTATTGTCCCTCGT |  |  |
| Mouse Ctla4  NM_005214.4 | CTACCTGGGCATAGGCAACG | 103 | 57.7 |
|  | CCCCGAACTAACTGCTGCAA |  |  |
| Mouse Prf1  NM_011073.3 | GAGCATCCCTGACTTTCCCTT | 84 | 56.4 |
|  | ATGTTTACGCTTCGTGGCAG |  |  |
| Mouse Gzmb  NM_013542.3 | GAAGCCAGGAGATGTGTGCT | 86 | 56.4 |
|  | GCTCAACCTCTTGTAGCGTG |  |  |

**S1 i**

CD69+

**
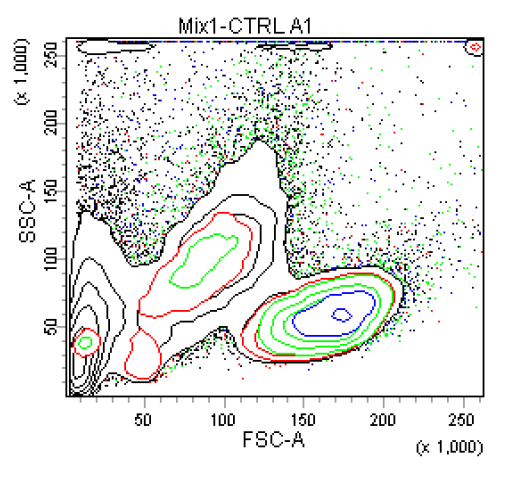
**

**ii**

**Figure S1. Gating strategies for CD4^+^/CD8^+^ T cell panels in murine splenocytes.** Stimulated splenocytes were stained for T cell markers CD45, CD4, CD8; activation marker CD69, and cytotoxic T-cell effectors Perforin and Granzyme B. Helper T-cells were identified as CD45^+^ vs CD4^+^ while Cytotoxic T-cells were identified as CD45^+^ vs CD8^+^. i) From the identified CD4^+^ T-cells, CD69^+^ cells were gated to determine activated CD4^+^ helper T-cells. ii) From the selected CD8 T-cells, dot plots for CD69 vs Perforin and CD69 vs Granzyme B were performed to determine activated cells expressing effector cytotoxic molecules.

**S2**


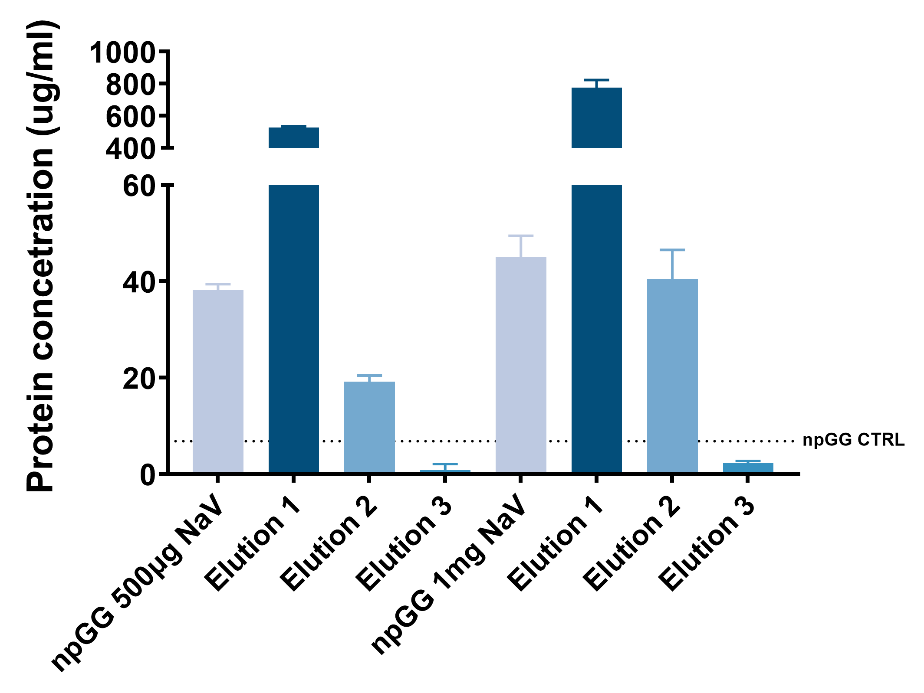


**Figure S2. Surface modification of npGG with NeutrAvidin.** NeutrAvidin density on npGG surface after functionalization with either 500 μg or 1 mg of NeutrAvidin determined by micro bicinchoninic acid assay. Elution’s obtained from each reaction were also assessed to determine unbound NeutrAvidin. Quantitative results are expressed as the mean ± standard deviation where n = 3.
